# In *Entamoeba histolytica*, a BspA family protein is required for chemotaxis toward tumour necrosis factor

**DOI:** 10.15698/mic2015.07.214

**Published:** 2015-07-06

**Authors:** Anne Silvestre, Aurélie Plaze, Patricia Berthon, Roman Thibeaux, Nancy Guillen, Elisabeth Labruyère

**Affiliations:** 1 Institut Pasteur, Unité Biologie Cellulaire du Parasitisme, F-75015 Paris, France.; 2 INSERM U786, F-75015 Paris, France.; 3 INRA, UMR1282 Infectiologie et Santé Publique, F-37380 Nouzilly, France.; 4 Université de Tours, UMR1282 Infectiologie et Santé Publique, F-37000 Tours, France.

**Keywords:** Entamoeba histolytica, chemotaxi, tumour necrosis factor, BspA protein

## Abstract

Background: *Entamoeba histolytica* cell migration is essential for the development of human amoebiasis (an infectious disease characterized by tissue invasion and destruction). The tissue inflammation associated with tumour necrosis factor (TNF) secretion by host cells is a well-documented feature of amoebiasis. Tumour necrosis factor is a chemoattractant for *E. histolytica*, and the parasite may have a TNF receptor at its cell surface. Methods: confocal microscopy, RNA Sequencing, bioinformatics, RNA antisense techniques and histological analysis of human colon explants were used to characterize the interplay between TNF and *E. histolytica*. Results: an antibody against human TNF receptor 1 (TNFR1) stained the *E. histolytica* trophozoite surface and (on immunoblots) binds to a 150-kDa protein. Proteome screening with the TNFR1 sequence revealed a BspA family protein in *E. histolytica* that carries a TNFR signature domain and six leucine-rich repeats (named here as "cell surface protein", CSP, in view of its cellular location). Cell surface protein shares structural homologies with Toll-Like receptors, colocalizes with TNF and is internalized in TNF-containing vesicles. Reduction of cellular CSP levels abolished chemotaxis toward TNF and blocked parasite invasion of human colon. Conclusions: there is a clear link between TNF chemotaxis, CSP and pathogenesis.

## INTRODUCTION

*Entamoeba histolytica* is the causative agent of human amoebiasis (a disease that targets intestinal and hepatic tissues). Parasite motility has an important role in invasive amoebiasis 1, since trophozoites cross the mucosal barrier and penetrate the intestinal epithelium. The motile trophozoites interact with extracellular matrix components and cells, leading to disruption of the intestinal architecture, cell death and acute inflammation 2.

During the host's amoeba-induced inflammatory response, human epithelial cells chemoattract neutrophils and macrophages to the site of invasion 3. Trophozoite motility is related to chemotactic stimuli derived from components such as activated C5α in human serum, lysed red blood cells, bacteria, N-acetylneuraminic acid, fibronectin, and human tumour necrosis factor (TNF) 4,5. However, the molecular interplay between these compounds and *E. histolytica* has not been extensively characterized.

The TNF secreted by enterocytes and macrophages is a major component in the amplification of amoeba-related inflammation 6. The levels of TNF secreted by the host is not sufficiently cleared through binding on trophozoites as this interaction does not influence the disease progress in experimental murine amoebiasis; only TNF depletion shows a reducing impact on the immunopathogenicity and the disease outcome 7. We showed that human TNF is chemoattractant and chemokinetic for *E. histolytica* 4,5,8. Chemotaxis of *E. histolytica* toward TNF is abrogated in presence of monoclonal anti-TNF antibody or with the soluble TNF receptor 1, demonstrating specificity of TNF chemotaxis 4. Transmembrane tumour necrosis factor receptors (TNFRs) are characterized by extracellular cysteine-rich domains that are the hallmark of the TNFR superfamily 9. To date, no members of the TNFR superfamily have been described in *E. histolytica*. Chemotaxis of *E. histolytica* 5,8 results in motility toward the chemoattractant source 4,10,11. The mechanism by which TNF induces *E. histolytica* chemotaxis is based on phosphatidylinositide-3-kinase signalling, reorganization of the actin-rich cytoskeleton and galactose/N-acetyl-galactosamine lectin activity 10. Our starting hypothesis was that TNF binds amoebic surface proteins capable of activating a signalling pathway involved in chemotaxis. Our goal was thus to determine whether *E. histolytica's* proteome contains TNFR homologues. In a BLAST search, we identified a protein presenting the TNFR signature and bearing six leucine-rich repeats (LRRs, structural motifs involved in protein interaction). Cell surface proteins with LRRs have already been identified as virulence factors in the anaerobic bacteria *Bacteroides forsythius* 12 and were named "*Bacteroides* surface protein A" (BspA). This type of protein has also been found in oral bacteria 13 and in anaerobic protozo

ans as a predicted consequence of lateral gene transfer from bacteria into protozoan genomes 14. It was shown that BspA-like proteins are involved in bacterial adherence, epithelial cell invasion 15,16 and fibronectin and fibrinogen binding 12. Direct evidence for an *in vivo* role of BspA in pathogenesis was provided by a study showing that BspA-defective bacteria were significantly less pathogenic than the wild type (WT) 17. In *E. histolytica*, the BspA-like family is composed of 116 proteins 18, one of which has been found at the plasma membrane of trophozoites 19. In the present work, we identified another member of the BspA-like family and named it "cell surface protein" (CSP). We found that CSP and TNF co-localized at the trophozoite surface; and are then internalized within vesicles. In order to gain insight into CSP's putative role in pathogenesis, we down-regulated expression of the *csp*-encoding gene by transfection of trophozoites with an antisense (AS) construct plasmid. Using biochemical and cellular analyses, we demonstrated that CSP has a key role in *E. histolytica's* ability to migrate toward a TNF gradient and to invade human colon explants. These findings suggest that CSP is significantly involved in TNF chemotaxis during the early stages of *E. histolytica* amoebiasis although the proof that CSP is a direct receptor for TNF is not established.

## RESULTS

### Identification of proteins with a TNFR signature

**Figure 1:**
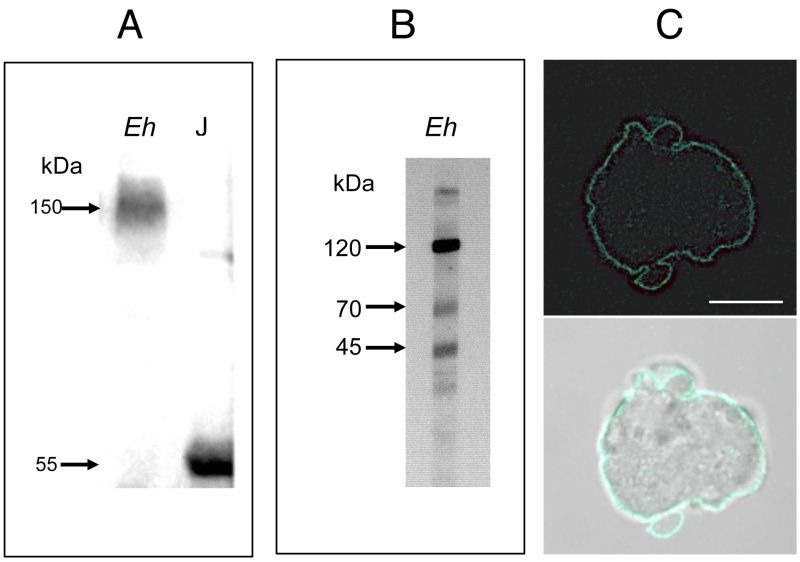
FIGURE 1: Screening of an E. histolytica protein extract with an antibody against human soluble hTNFR1. **(A) ** Polyacrylamide gel electrophoresis under non-reducing conditions, and a Western blot analysis. Crude lysates of *E. histolytica* (Eh) and Jurkat (J) cells were loaded in the indicated lane (load: 20 µg of protein). Probing with the anti-hTNFR1 antibody revealed a single 150 kDa protein in E. histolytica and a single 55 kDa protein in Jurkat cells. **(B) ** Polyacrylamide gel electrophoresis under reducing conditions, and a Western blot analysis. In a crude lysate from *E. histolytica*¸ three proteins (at approximately 120 kDa, 70 kDa and 45 kDa) were detected. (C) Entire trophozoites were fixed and stained with goat anti-hTNFR1 and anti-goat AlexaFluor488 in order to reveal the localization of amoebic proteins sharing an epitope with hTNFR1. Scale bar: 10 µm.

In order to determine whether proteins within the *E. histolytica* proteome shared common epitopes with human TNFR, a crude lysate from *E. histolytica* trophozoites was immunoblotted with an antibody against hTNFR1. A protein with a relative molecular mass of 150 kDa was detected by electrophoresis under non-reducing conditions (Figure 1A); whereas three proteins (120 kDa, 70 kDa and 45 kDa) were found upon treatment with reducing agents (Figure 1B). A crude lysate from Jurkat cells was used as a positive control; as expected, a signal at 55 kDa (corresponding to hTNFR1; accession number: AAA36754, 20) was detected (Figure 1A). To determine the cellular localization of the homologous amoebic proteins, parasites in culture were fixed and stained with an anti-hTNFR1 antibody (Figure 1C). Labelling was observed at the surface of non-permeabilized trophozoites, suggesting that an *E. histolytica* surface protein shared a common epitope with the hTNFR1.

**Figure 2:**
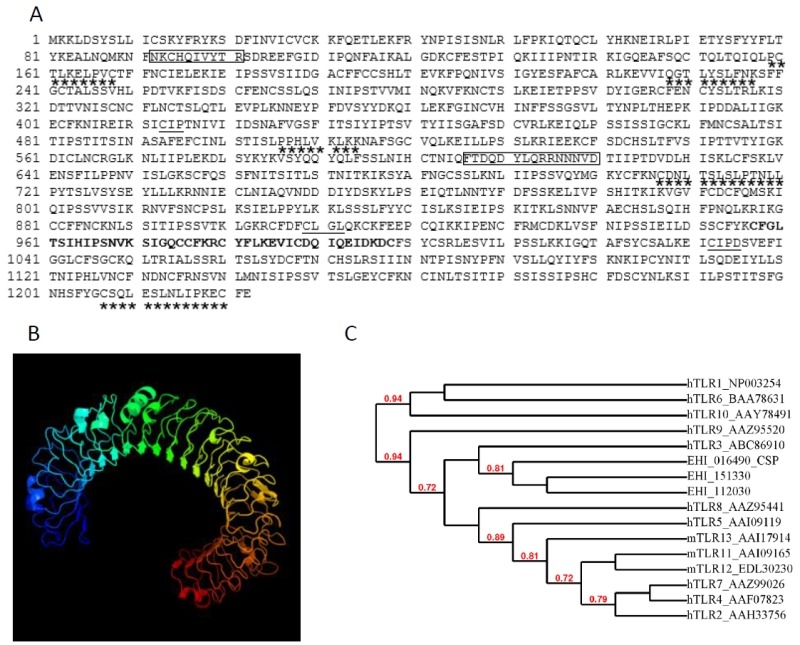
FIGURE 2: In silico analysis of the CSP (EHI_016490) amino acid sequence. **(A)**Protein domains found in the amino acid sequence of EHI_016490 after scanning with InterProScan and SMART online tools. The TNFR_NGFR_1 family's cysteine-rich region signature is indicated in bold. The leucine-rich repeats (xxxLxxLxx) are underlined with stars, the prenylation sites (CaaX, where each “a” is an aliphatic amino acid) are underlined and the two peptides used to produce polyclonal rabbit serum are bordered. **(B) **A three-dimensional structure model of EHI_016490, generated using Phyre2. The model is based on the c1ziwA template (the structure of the human toll-like receptor 3 extracellular domain). In all, 856 residues (70% of the full sequence) were modelled with a confidence score of 99.9% for the highest-scoring template. (C) Dendogram showing the relatedness between EHI_016490, EHI_112030, EHI_151330 from * Entamoeba histolytica *, human TLR (hTLR) and murine TLR (mTLR). The number at each branch point represents percentage bootstrap support, calculated from 1000 replicates. Bootstrap values below 0.7 are not represented.

The amino acid sequence of hTNFR1 was used in a BLAST search of the amoebic proteome in the AmoebaDB. Of 8201 ORFs representing the amoebic proteome, a single sequence was identified (E-value: 3.5x10-7; entry EHI_016490; predicted molecular mass: 140 kDa) as comprising a TNFR domain (Figure 2A). EHI_016490 is annotated in AmoebaDB as a member of the LRR BspA-like family and we named it "cell surface protein" (CSP). The putative protein contains 1222 amino acids, shares 43% amino acid sequence identity with hTNFR1 (E-value=0.74) and does not contain a conventional signal peptide or a transmembrane domain. SignalP 4.1 server (used to predict the presence and location of signal peptide cleavage sites in amino acid sequences from Gram-positive prokaryotes, Gram-negative prokaryotes, and eukaryotes) was employed and conventional signal peptides were not found in CSP sequence, but we cannot rule out the fact that CSP amino acid sequence might contain a signal peptide different from those described for other organisms. Prosite predicted a cysteine-rich TNFR/nerve growth factor receptor (NGFR) domain between amino acids 957 to 998 (PS00652, TNFR_NGFR_1 family cysteine-rich region signature). Six LRR5 domains (Pfam13306, with E-values ranging from 4.03 x 10e-29 to 9.6 x 10e-17) were found. LRR5 domains account for 61% of the total CSP protein. Two of the domains show near-perfect identity with the consensus sequence CxxLxxLxLxxxL (at positions 707 and 1207), whereas four are degenerated sequences (at positions 162, 231, 509 and 1060). Three predicted prenylation sites (CaaX, where “a” is an aliphatic amino acid) were found (at positions 413, 909 and 1032, respectively). We used the Phyre2 server to predict alpha helix, beta strand and coiled domains with the CSP amino acid sequence and thus gain insight into the protein's secondary structure. The best score was obtained for the human extra cellular domain of toll-like receptor 3 (TLR3) 21 (Figure 2B), as characterized by a confidence score of 99.9% for 70% of CSP's total sequence and a secondary structure prediction score of 7.7 (with a peak at 9).

Further analysis on CSP structure was performed using I-TASSER 22 algorithms. The best structural prediction for CSP fit with the identified mouse TLR3 ectodomain (Accession number: 3ciy in Protein Database Bank) (supplemental Figure S1). The prediction emerge with a high confidence score (c-score = -1.5 from a theoretical value ranging from -5 to +2), indicating that more than 90 % of the predicted model was correct. A TM-score over 0.5 (theoretical value ranging from 0 to 1) supporting in addition the high quality of the structural alignment between CSP and known structures for TLR3 in the Protein Database Bank.

Because CSP showed high homology with TLRs, we investigated whether the entire BspA like family from *E. histolytica* (to which CSP belongs) has phylogenetic relatedness to TLR family of receptors. To identify the genes from the BspA family expressed in cultured *E. histolytica*, we took advantage of our precedent RNA seq approaches performed with *E. histolytica* virulent strain 23. A total of 95 genes encoding BspA like proteins (over 100 formerly identified) were transcribed (Supplemental Table S1). To determine the genetic relatedness of CSP (EHI_016490) with its *Entamoeba* BspA like protein orthologues, a phylogenetic tree was build considering the 95 BspA predicted protein sequences from *E. histolytica* genome (supplemental Figure S2). The order of the sequences in the alignment reflects their relatedness with the most similar sequences typically being aligned next to each other. According to the topology of the evolutionary tree, EHI_016490 diverged early from the 94 other BspA like orthologues found in *E. histolytica*. Phylogeny of two other candidates (EHI_112030 and EHI_151330) was closely related to CSP (EHI_016490).

To gain insight into phylogenetic relatedness between CSP and mammalian TLR family of receptors, another phylogenetic tree was built with amino acid sequences from CSP (EHI_016490), its orthologues EHI_112030 and EHI_151330, human TLR (10 hTLR) and murine TLR (3 mTLR) (Figure 2C). The 16 sequences were dispatched in three distinct clusters, supported by high bootstrap values (72-94 %). The tree topology confirmed that CSP and its two closely related BspA like orthologues from *E. histolytica* (EHI_112030 and EHI_151330) were closely related to human TLR3.

Confronting EHI_112030 and EHI_151330 with PDB structural entries (using Phyre2 server) allowed proposing their secondary structure which showed similarities to these proposed for CSP: the top ten highest scoring alignments were with TLR domains. For the two-tested BspA like proteins, the best score was found with the extra cellular domain of human TLR3 (supplemental Figure S3). All the data from the bioinformatics analysis lead us to though that the TLR-like structure in CSP was mainly generated by LRR motifs, these represents 61 % of the total protein. As a consequence, we can expect that the distant prioritized BspA like proteins in the tree (also bearing LRR motifs) should have the same TLR 3D structure. Phyre 2 structural approaches performed on the two most distant proteins tell us that it was not the case: EHI_072070, EHI_152950 do not have the classical horseshoe-like structure of TLRs (supplemental Figure S3). For these two last candidates, the best score (100 % alignment) fit with a leucine rich protein 2 from *Bacteroides ovatus*, with coverage of 82-86 %. Altogether, our bioinformatics analysis defined structural specificity of CSP and two orthologues among the large BspA like proteins family from *E. histolytica* and described homologies with TLRs from humans.

### The amoebic CSP binds TNF and relocates to the uropod when a TNF gradient is present

In order to localize the CSP in the trophozoite, two potentially immunogenic peptides (Phe-92 to Glu-106 and Phe-606 to Thr-621) were synthesized and used to produce anti-CSP antibody. Probing *E. histolytica* crude lysates with the anti-CSP serum revealed three bands at 140, 70 and 45 kDa (supplemental Figure S4). CSP was predicted to be a roughly 140 kDa protein, which is the apparent molecular mass corresponding to the higher signal. The two smaller peptides recognized in reducing gel conditions might represent processed forms of CSP as we have already observed with the anti-sTNR human antibody (Figure 1B).

**Figure 3:**
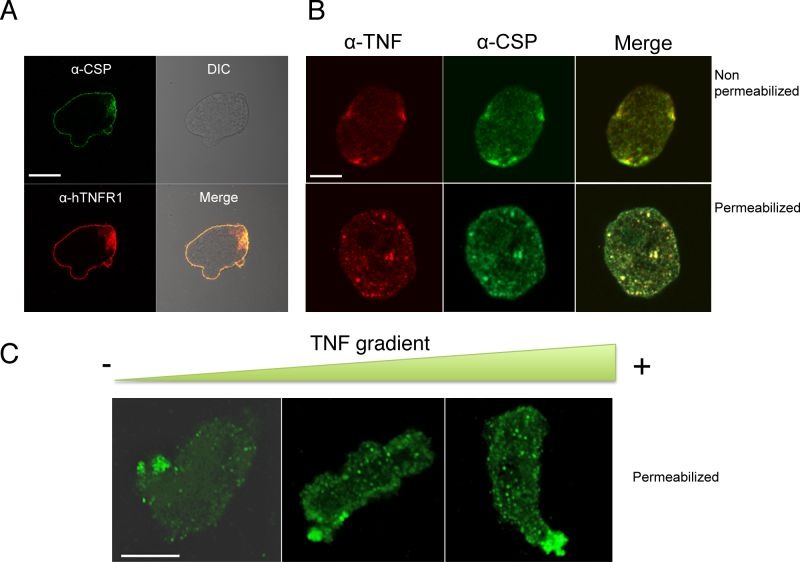
FIGURE 3: Cellular localization of CSP in Entamoeba histolytica. **(A) **Immunolocalization of CSP (green) and hTNFR1 (red) in fixed but non-permeabilized trophozoites revealed that both proteins are located at the plasma membrane. Scale bar: 10 µm. **(B) ** Immunolocalization of CSP in *E. histolytica *incubated with a homogenous (non-gradient) concentration of TNF. Trophozoites were incubated with 70 nM TNF, fixed and permeabilized or not. CSP (green) and TNF (red) were co-localized at the trophozoite surface. CSP was detected as described for Fig. 3A. TNF was detected with 1E12 monoclonal antibody and mouse-Cy3 (dilution: 1:200). Scale bar: 10 µm. **(C) ** Immunolocalization of CSP in *E. histolytica * incubated with a TNF gradient. Trophozoites exposed to a TNF gradient for 2 h were fixed and permeabilized. During trophozoite migration toward the TNF source, CSP (green) concentrated in the rear part of the cells (i.e. distal to the TNF source). The nucleus was stained with DAPI (blue). Scale bar: 10 µm.

Anti-CSP and anti-TNFR antibodies were used to examine the proteins in trophozoites, using confocal microscopy. After staining trophozoites with both antibodies, the fluorescence signals were seen to co-localize in a continuous pattern on plasma membrane (Figure 3A). A control with pre-immune serum gave a low background signal and no signal at the trophozoite surface (data not shown). When trophozoites were incubated with recombinant human TNF and labelled with anti-CSP and anti-TNF antibodies, the trophozoite surface was stained by small dots of labelling, as a patched pattern. The fluorescence signals co-localize at the cell surface - showing that CSP and TNF bind to each other. When trophozoites were permeabilized, the fluorescence signals still co-localized as intra-cytoplasmic vesicules of various sizes (Figure 3B).

To investigate the potential influence of TNF on CSP localization during chemotaxis, trophozoites were incubated in a TNF gradient. CSP was detected in intracytoplasmic vesicles when the trophozoites were far from the TNF source but was concentrated in the uropod when the cells were close to the source (Figure 3C). These results indicate that CSP is usually located at the trophozoite surface but relocates to the uropod when trophozoites migrate up a TNF gradient. Several attempts were performed trying to express *csp* encoding gene in *Escherichia coli* to further analyze the physicochemical parameters of the possible interaction between TNF and CSP. Unfortunately, we did not succeed in cloning the entire gene into the bacterium, suggesting that this gene maybe toxic for *E. coli*.

### Knocking down CSP with an AS RNA technique 

**Figure 4:**
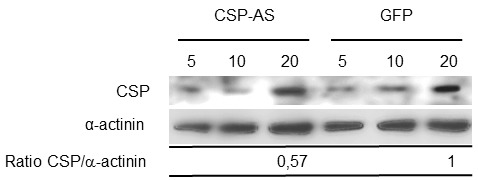
FIGURE 4: Quantification of CSP expression in modified *Entamoeba histolytica* trophozoites. Five, ten and twenty µg of protein from crude trophozoite extracts were loaded. CSP (140 kDa) and actinin (70 kDa) were revealed with specific antibodies. A decrease in CSP abundance was observed in trophozoites expressing the CSP-AS construct. Amounts of CSP and actinin in 20 µg of crude extract were quantified using AlphaEaseFC software. Levels of CSP were normalized against the amount of α-actinin. The relative abundance of CSP was 57% lower in CSP-AS trophozoites than in WT and GFP trophozoites (P = 0.04). Data are representative of three independent experiments.

To examine CSP's possible role in chemotaxis toward TNF, trophozoites were transfected with a plasmid carrying the *csp* gene inserted in an antisense orientation. The effect of CSP-AS RNA transcription on CSP levels was detected in immunoblot experiments (Figure 4); the level was 43% lower in CSP-AS trophozoites than in WT trophozoites (n=3; p=0.01). There was no significance between WT trophozoites and control GFP trophozoites in terms of protein levels of CSP.

### The functional role of CSP in chemotaxis toward TNF

**Figure 5:**
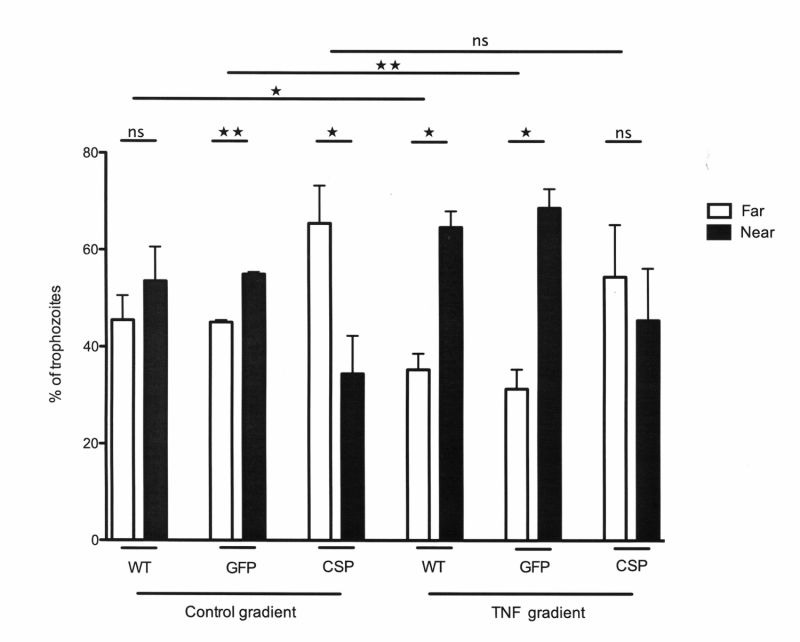
FIGURE 5: In vitro TNF chemotaxis is abolished in CSP-AS trophozoites. The displacement of the *E. histolytica* trophozoites was analyzed after 2 h of incubation in the presence of various compounds. Mean numbers of trophozoites in each group (either distal or proximal to the source) were compared in a Student's t-test. In the absence of chemoattractant (incomplete TY medium), WT trophozoites were distributed homogeneously along the coverslip. When the agarose slice was filled with TNF (50 nM), there were significant displacements of WT and GFP trophozoites up the gradient toward the TNF source. In contrast, the presence or absence of a TNF gradient did not significantly affect the distribution of CSP-AS trophozoites. Data are representative of three to four independent experiments. ** P < 0.01; * P < 0.05; ns, not significant.

To determine whether CSP is involved in chemotaxis toward TNF, the displacement of trophozoites in which CSP expression had been modified was investigated *in vitro*. In the absence of chemoattractant, WT trophozoites were evenly distributed along the coverslip (p=0.531) (Figure 5). When TNF was injected into the agarose slice, trophozoites were significantly more abundant near the source of TNF when WT (p=0.042) and GFP (p=0.005) strains were tested. In contrast, similar numbers of CSP-AS trophozoites were located near to or far from the TNF source (p=0.249), indicating that most of these cells did not react to the presence of TNF. These results suggest that CSP is involved in TNF chemotaxis in trophozoites.

### CSP is important for spreading within the human intestine

We used an *ex vivo* human model of intestinal amoebiasis to further investigate CSP's role during intestinal invasion by *E. histolytica*, 24. This model has already enabled us to describe the early stages of pathogen invasion, including TNF secretion within the first 4 h of amoebic invasion 24.

**Figure 6:**
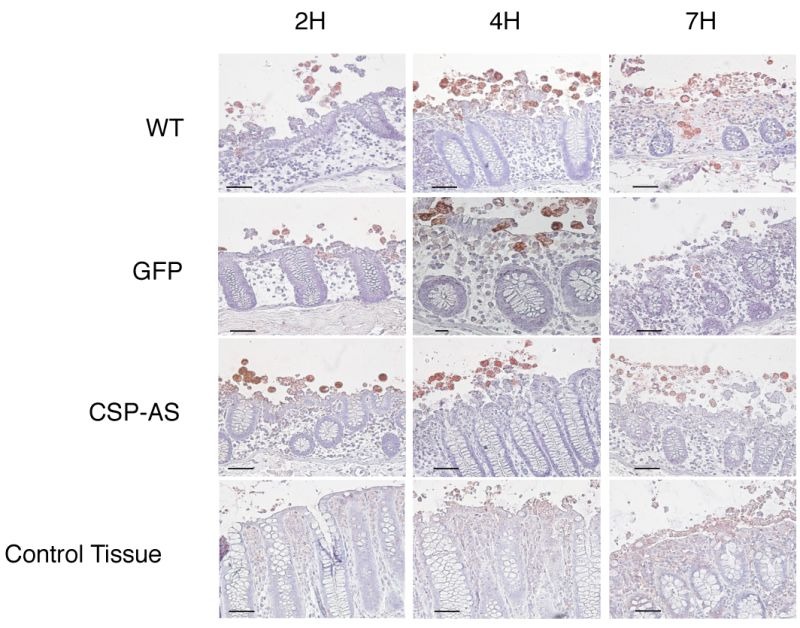
FIGURE 6: Invasion of ex vivo colonic explants was impaired for CSP-AS trophozoites. Histological examination of colonic tissue sections after 7 h of infection with *E. histolytica*WT, GFP, CSP-AS strains and in the absence of trophozoites (control). Tissue cross-sections were stained with hematoxylin/eosin reagent. Trophozoites were revealed with an antibody against Gal/GalNAc lectin and appear in red. WT and GFP trophozoites were able to invade the mucosa. In contrast, trophozoites expressing the CSP-AS construct were unable to invade the lamina propria and tended to remain at the surface of the mucosa. Images are representative of three individual experiments. Scale bar: 10 µm.

Prior to infection, the viability of CSP-AS trophozoites in Krebs' buffer was determined in a trypan blue exclusion test. The mean ± SD percentages of dead cells were similar for GFP-expressing trophozoites and CSP-AS trophozoites (8.27 ± 0.56% and 8.40 ± 0.73%, respectively). Next, CSP-AS, WT and GFP trophozoites were incubated with human colon explants for 2, 4 and 7 h. Trophozoites were then detected on histological sections by immunostaining the Gal/GalNAc lectin (Figure 6). After 2 h of incubation, WT and GFP trophozoites adhered to the colonic epithelium. After 4 h, trophozoites detached the enterocytes and migrated into the lamina propria along the crypts. After 7 h of incubation, the tissue architecture was altered and trophozoites were found deep in the lamina propria, as observed in previous studies 24,25,26,27. The destruction of the mucosa by WT and GFP trophozoites contrasted with the unaltered architecture of the control tissue after 7 h of culture.

The CSP-AS trophozoites differed from the WT in terms of intestinal invasion. Although no differences in tissue penetration were observed during the first 2 h trophozoites, the CSP-AS trophozoites' impairment in the invasive process appeared after 4 h; the colonic mucosa had not been invaded at this time point. Even after 7 h of incubation, CSP-AS trophozoites were never found into the lamina propria. Although some epithelial cells were detached, the overall tissue architecture was unaffected. The time point for the CSP-AS phenotype coincided with the TNF secretion previously observed after 4 h of explant incubation with *E. histolytica* (29). Taken as a whole, these data suggest that CSP has a role in amoebic chemotaxis toward TNF during the early steps of intestinal invasion.

## DISCUSSION

During human intestinal invasion, *E. histolytica* trophozoites degrade the mucus layer, adhere to epithelial cells, lyse cells and penetrate deep into the mucosa. Several parasitic proteins are known to be involved into these crucial events, i.e. the cysteine proteinase involved in mucus and extra cellular matrix degradation 28, the Gal/GalNAc lectin involved in cells and mucus adhesion and the amoebapore involved in cell death (for a review, see 29). However, trophozoites with impaired Gal/GalNAc lectin signalling and impaired amoebapore A expression are still able to invade the human intestinal mucosa 24. Trophozoites with impaired cysteine proteinase CP-A5 expression cross the mucus layer but are unable to penetrate deep into the mucosa, due to a lack of collagen remodelling via host matrix metalloproteinase activation 25. None of these defective amoebic strains are impaired in cell adhesion or mucus adhesion 24. In contrast, trophozoites with low levels of β-amylase are unable to deplete the mucus layer and thus unable to invade colonic explants 27. In the present study, we characterized CSP (EHI_016490) and found that it is involved in tissue invasion by *E. histolytica* and chemotaxis toward TNF but not in mucus layer degradation.

Chemotaxis consists in the sensing of a chemoattractant molecule by cell surface proteins. The subsequent signal transduction leads to changes in the dynamics of the actin-rich cytoskeleton, which in turn induce trophozoite polarization 30. These morphological changes result in directed cell movement.

The first step in TNF chemotaxis relies on TNF sensing by amoebic surface proteins. Our indirect immunofluorescence assay revealed that CSP was found associated to the trophozoite plasma membrane. However, the CSP sequence does not contain a conventional signal sequence and is not predicted to include a transmembrane domain, several other *E. histolytica* proteins (including the lysine- and glutamine-rich protein KERP1, peroxiredoxin and a BspA-like protein EHI_013940) lacking these features have been also found at the plasma membrane of trophozoites 19,31, the mechanism of their traffic is not yet elucidated.

CSP might be bound to the *E. histolytica* membrane through a prenyl lipid anchor (as it has been suggested for other BspA-like proteins). Prenylation has not been described as a modification allowing protein anchoring to the external part of the plasma membrane. In contrast, the attachment of hydrophobic prenyl groups can anchor the proteins to intracellular membranes. Based on the power of genomics and computer predictions, our hypothesis is that following prenylation, CSP may bind ER-membranes and undergo CAAX box (Cys-aliphatic-aliphatic-Xaa) processing (CSP's sequence contains three prenylation sites, including two at the carboxyl terminus) with the cleavage of -AAX and carboxymethylation of the prenylated Cys residue. The eventual lipid modifications on CSP may favor the transport to the cell surface by vesicular trafficking, but this remains to be determined. Our hypothesis is supported by the detection of isoprenylation activity in *E. histolytica* 32,33.

To determine the role of prenylation in anchoring CSP to the plasma membrane, it is possible to inhibit the enzymes necessaries for this phenomenon and that has been efficient in several protozoan parasites such as *Trypanosoma brucei*, *Leishmania mexicana*, *Plasmodium falciparum* 34,35,36. Lastly, it is possible to inhibit vesicular trafficking with drugs such as Brefeldin A.

Although CSP seems to be devoid of transmembrane domain, CSP may acts as a receptor, in association with ancillary proteins (with transmembrane domains) which clustering with CSP would lead to TNF chemotaxis signaling. We showed that anti-CSP antibody stained the trophozoite surface, and TNF binding occurs upon addition into the extracellular milieu of this molecule, both data support that at least a CSP moiety is present at the trophozoite surface. Moreover, CSP shows a uniform distribution on the plasma membrane of the trophozoite, and in presence of TNF, CSP distribution becomes patched. This may correspond to clustering of CSP induced by TNF.

The second step in chemotaxis consists in the transduction of the surface signal toward the intracellular cytoplasm, leading to changes in the trophozoite's actin-rich cytoskeleton. Phosphatidylinositol 3,4,5-triphosphate and phosphatidylinositol 3-kinase (PI3K) have emerged as important components of the chemotactic signaling pathway in eukaryotic cells 37. Previous work has demonstrated that *E. histolytica* chemotaxis toward TNF is abrogated after treatment with the PI3K inhibitor wortmannin 10. Recently, it was shown that wortmannin directly affects amoebic motion and *in vivo* virulence by altering the structure of the cytoskeleton 38. The effect of TNF signaling on amoebic gene expression has been analyzed in microarray experiments. Transcription of genes encoding proteins involved in cytoskeleton dynamics (microfilament dynamics, nucleation and bundling) was found to be modified 10,39. The third and last step in chemotaxis consists in the morphological changes that result in directional displacement of the cell.

CSP is a unique BspA-like amoebic protein that possesses a TNFR domain and a LRR motif (possibly involved in fibronectin/fibrinogen binding 12) and is essential for invasion of the human colonic mucosa. The taxonomic distribution of genomes encoding BspA-like proteins indicates that most are bacterial lineages found in the gut of vertebrates, including humans 40. Furthermore, BspA-like proteins have been found in three unicellular protozoans: *E. histolytica*, *E. dispar* 19 and *Trichomonas vaginalis* 40. It was suggested that lateral gene transfer (LGT) from bacteria was responsible for BspA-like acquisition by the three protozoans. Indeed, LGT in *E. histolytica* genome may confer a direct, adaptive advantage on this species, since the functions encoded by the transferred gene should circumvent some constraints of the anaerobic, parasitic lifestyle. This hypothesis is supported by the fact that many amoebic genes encoding metabolic and fermentation enzymes (e.g. β-amylase) are derived from prokaryotes 41. A growing body of evidence indicates that the donor organism may belong to the *Cytophaga-Flavobacterium-Bacteroides* group of the phylum *Bacteroidetes* 40,42,43. The sequence of the CSP-encoding gene supports the hypothesis of CSP acquisition via LGT from a *Bacteroidetes*-group donor present in the commensal flora (microbiota) of the human gut.

We found that CSP had the same secondary structure as the ectodomain of the human toll-like receptor (TLR) 3. Toll-like receptors are sensors evolutionarily conserved between plants, fly and mammalians. TLRs recognize danger signals from diverse array of pathogens, which are called pathogen-associated molecular patterns (PAMPs), and also recognize molecules (including nucleic acids) released from intracellular stores of damaged or stressed cells, for review 44,45. TLR family has expanded greatly over the last few years, with 13 TLR now described, 10 of which are found in humans. All TLRs are composed of an ectodomain containing multiple LRRs directly involved in the recognition of PAMPs, a TM domain required for the subcellular localization of TLRs, and an intracellular domain with a conserved cytoplasmic signaling region called the Toll/IL-1 receptor (TIR), that triggers downstream complex signalling networks required to sustain appropriate immune response. In mammalian cells, TLRs recognize bacterial surface or cell wall components such as lipopolysaccharide, lipoteichoic acid and peptidoglycan 46. The typical TLR (consisting of a LRR ectodomain and a cytoplasmic Toll/interleukin-1 receptor (TIR) domain) may result from the combination of a LRR-only protein and a TIR-only protein 47. In this context, CSP may correspond to an ancestral (TIR-negative) form of the TLR present in higher eukaryotes.

In conclusion, we showed that CSP is located at trophozoite surface, the role of prenylation remains to be demonstrated. We hypothesize that it is associated to ancillary proteins with transmembrane domain to lead to TNF chemotaxis signaling. CSP-AS trophozoites may have low amoebic pathogenicity because they were impaired in invasion of the colonic mucosa. Further work is needed to characterize the interplay between CSP and the signaling pathway involved in TNF chemotaxis.

## MATERIALS AND METHODS

### Ethical aspects 

Human intestinal tissues were processed according to the French Bioethics Act, following approval from two investigational review boards (Comité de Protection des Personnes Ile de France VII and Institut Pasteur Recherche Biomedicale: reference RBM/2009.50).

### Amoebae strains and culture conditions

The HM1-IMSS pathogenic *Entamoeba histolytica* strain was cultured axenically in complete TYI-S-33 medium 48 at 37°C. Trophozoites were harvested during the exponential growth phase, collected by centrifugation at 1000 g for 10 min at 4°C, and then washed thoroughly with either complete TYI-S-33 or phosphate buffered saline (PBS) to remove all nutrients. Incubation with 75 nM human recombinant TNF (R&D Systems) was performed with 1.5 x 10^6^ amoebae, in complete TYI-S-33 medium and at 37°C. The amoebae were then washed in PBS. Jurkat cells were cultured in RPMI (Gibco) at 37°C.

### Plasmid construction and trophozoite transfection

An RNA AS technique was used to reduce CSP expression in a stable manner. The pEhNeo/CSP-AS plasmid was constructed by replacing the chloramphenicol acetyltransferase (CAT) coding sequence of pEhNEO/CAT 49 by the CSP AS (EHI_016490) sequence (generated by PCR using total RNA from trophozoites as a template). The primers were as follows: EhCSP1 5’ GATCGGATCCTATTCGTTATTGATTTGTAGTAAATATTTTCGATATAAAAG 3’ and EhCSP2 5’ GATCGGTACCGATAACTAACTTTATATTTATATGACAAATCTTTTTCTAATG 3’. The PCR product was cloned, sequenced and subcloned into the KpnI and BamHI restriction sites of pEhNEO/CAT, flanked by untranslated 5’ and 3’ sequences of *E. histolytica* actin gene. The control transfection vector (pEhNEO/GFP) was constructed by cloning of GFP in the unique BamHI site of the pExEhNeo plasmid. GFP sequence was generated with primers: GFP1 5’ GGATCCATGAGTAAAGGAGAAG 3’ and GFP2 5’ TCATAGGATCCGGGTATCTTGA 3’ 50. After electroporation, transfected cells were isolated in the presence of G418 (30 µg/ml), as described previously 51.

### Immunofluorescence staining of amoebae

The entire immunofluorescence procedure was carried out at 37°C, unless otherwise indicated. Amoebae were fixed in 3.7% paraformaldehyde for 30 min, permeabilized by adding 0.1% Triton X100 in PBS for 2 min at room temperature, and blocked in 1% bovine serum albumin in PBS for 30 min. Preparations were then incubated with the following antibodies: anti-CSP (I8, a custom rabbit polyclonal antibody raised against the peptides FNKCHOIVYTRSDREEE and FTDQDYLQRRNNNVDT (Eurogentec, Belgium)) and anti-TNF (1E12, anti-TIP domain, a kind gift from Dr Etienne Pays, Université Libre de Bruxelles, Belgium) 52. Secondary antibodies coupled to AlexaFluor488 or AlexaFluor546 (Molecular Probes) were added (dilution 1:200) for 30 min at 37°C. The samples were mounted in Prolong medium containing DAPI (Invitrogen). Amoebae were examined by confocal microscopy (LSM510, Zeiss).

### Western blot analysis

To evaluate the TORC1-dependent C-terminal phosphorylation Crude trophozoite extract (10-20 µg) was separated on 10-12% polyacrylamide gels, transferred onto nitrocellulose, and blocked with 5% dry milk in Tris-buffered saline/Tween 20. Proteins were probed with a goat anti-hTNFR1 (Sigma) (dilution 1:1000) and the anti-CSP (dilution 1:1000). Detection was performed with an anti-rabbit antibody (dilution 1:10000) (Jackson ImmunoResearch) and enhanced chemiluminescence reagents (Amersham Biosciences). Detected proteins were quantified using AlphaEaseFC software (Alpha Innotech).

### *In silico* protein analysis

The protein sequence of human TNFR1 (hTNFR1; GenBank reference AAA36754 20) was used to perform a BLASTp search of the *E. histolytica* AmoebaDB database (http://amoebadb.org/amoeba). Signal peptide was predicted with SignalP algorithm 53, and transmembrane helix domains were predicted with the TMHMM-2.0 algorithm 54. Post-translational modifications were identified with Prosite 55. Conserved protein domains were identified using the PFAM 56, InterProScan 57 and SMART 58 online tools. Protein structure was predicted with the protein homology/analogy recognition engine (Phyre2) 59. Phyre2 uses a library from the Structural Classification of Proteins (SCOP) Database, augmented with new depositions in the Protein Data Bank (PDB). Briefly, Psi-Blast was run to collect homologous sequences and built a statistical sequence profile. Three independent secondary structure prediction programs (Pri-Pred, SSPro and JNet) were run, to generate a final consensus prediction. The confidence of the secondary structure prediction was determined by a prediction score (theoretical value ranging from 1 to 9). The highest the score, the highest the accurate secondary structure prediction. The top ten highest scoring alignments were used to construct full 3D models of the query. Phyre2 displayed the 3D models of the query, with the alignment coverage, the identity percentage and the confidence of the model. Phyre2 allows achieving high accuracy models at very low sequence identities (15-20 %).

The second freely available web server used for structure prediction was the iterative threading assembly refinement I-Tasser 22,60. I-TASSER fragments the query sequence into overlapping short stretches of amino acids. Candidate structures for those small fragments are generated as mentioned earlier, and assembled to construct a low-energy protein conformation.

A phylogenetic analysis was run on 95 Bsp A like proteins found in *E. histolytica* genome and expressed in trophozoites, to determine the genetic distance between EHI_016490 and its orthologs. The multiple alignment of 95 BspA like amino acids sequences was obtained by using ClustalX software 61. The Neighbor-Joining algorithm was used to infer the topology based on multiple sequences alignment with identity percentage distance.

### *In vitro* TNF chemotaxis

To determine whether TNF chemotaxis was altered in *E. histolytica* trophozoites expressing the CSP-AS construct, we used the agarose chemotaxis-on-coverslip assay 4. The coverslips were mounted onto glass slides and the cell distribution was examined under the microscope. Trophozoites were classified arbitrarily as groups that were near to or far from the source of test compound.

### Preparation of human colon explants 

Segments of human colon were obtained anonymously from three fully informed patients undergoing colon surgery. The conditions used for handling human colon explants have been published elsewhere 24. Trophozoites (8x10^5^) were added to the luminal face of the colon and incubated in Krebs' medium at 37°C for 2, 4 and 7 h. Amoebae-free segments served as controls for each time point.

### Histological analysis

Tissue invasion by *E. histolytica* was monitored after incubation. Tissues were fixed in 4% paraformaldehyde in PBS for 48 h at 4°C and then embedded in paraffin. Three sections (thickness: 5 µm) were cut from paraffin blocks and stained with standard hematoxylin/eosin reagent. Trophozoites were stained with a 1:200 polyclonal antibody raised against the Gal/GalNAc lectin 62. For each experiment, a representative histology image was taken.

### Statistical analysis 

Intergroup differences were evaluated in Student’s unpaired t-test, using GraphPad software (http://www.graphpad.com). The threshold for statistical significance was set to P = 0.05.

## SUPPLEMENTAL MATERIAL

Click here for supplemental data file.

All supplemental data for this article are also available online at  http://microbialcell.com/researcharticles/in-entamoeba-histolytica-a-bspa-family-protein-is-required-for-chemotaxis-toward-tumour-necrosis-factor/.
