# Supplementary material for: In Entamoeba histolytica, a BspA family protein is required for chemotaxis toward tumour necrosis factor
Source: Microb Cell. 2015 Jul 6;2(7):235–46. doi: 10.15698/mic2015.07.214 (PMC5349171; doi:10.15698/mic2015.07.214)
Supplement: Supplementary file 1 [file mic-02-235-s01.pdf]

The following supplemental information is available for this article:

Table S1. RNA seq data of BspA like gene transcription from virulent strain of *E. histolytica.*

	Unicity	Number of reads	Number of reads gene size corrected (read per kb per million mapped reads)	Gene length (bp)
EHI_000760	1	255	2,006	1202
EHI_002120	1	101	1,49	641
EHI_003380	1	25	0,278	851
EHI_003790	1	3	0,059	482
EHI_004360	1	605	3,233	1769
EHI_008340	1	1	0,022	428
EHI_010000	1	44	0,383	1085
EHI_012090	1	264	3,787	659
EHI_013750	1	2	0,023	812
EHI_013940	1	125	0,66	1790
EHI_015120	1	30084	281,587	1010
EHI_016240	1	1	0,007	1342
EHI_016490	1	561	1,446	3668
EHI_017710	1	551	4,069	1280
EHI_020090	1	133	0,557	2258

EHI_024580	1	425	3,491	1151
EHI_027680	1	407	2,978	1292
EHI_028330	1	12	0,243	467
EHI_034610	1	125	1,111	1064
EHI_038810	1	1388	6,712	1955
EHI_041470	1	2	0,014	1346
EHI_042470	1	11	0,19	547
EHI_046800	1	5	0,098	482
EHI_047820	1	1	0,01	926
EHI_048660	1	244	1,064	2168
EHI_049160	1	8	0,062	1217
EHI_051070	1	150	0,818	1733
EHI_051080	1	328	1,037	2990
EHI_054160	1	1405	15,128	878
EHI_062750	1	116	0,924	1187
EHI_063030	1	846	9,636	830
EHI_064920	1	2	0,04	467
EHI_069190	1	2079	15,391	1277
EHI_070230	1	190	1,323	1358

EHI_070330	1	10	0,065	1454
EHI_072070	1	1995	14,7	1283
EHI_075960	1	705	4,132	1613
EHI_077280	1	2	0,011	1670
EHI_078570	1	402	2,208	1721
EHI_079970	1	8	0,072	1050
EHI_082060	1	52	0,248	1985
EHI_084160	1	5	0,079	596
EHI_088100	1	1590	12,412	1211
EHI_094080	1	207	0,772	2534
EHI_095060	1	656	4,789	1295
EHI_100700	1	18	0,115	1478
EHI_102380	1	19	0,168	1067
EHI_103140	1	16	0,194	778
EHI_105370	1	1	0,012	769
EHI_107220	1	237	1,907	1175
EHI_110590	1	603	2,252	2531
EHI_110760	1	73	0,508	1358
EHI_111950	0	0	0	580

EHI_111960	1	6	0,115	494
EHI_112030	1	235	0,661	3359
EHI_112290	1	2	0,03	640
EHI_113310	1	9	0,161	527
EHI_113990	1	2798	7,903	3347
EHI_115500	1	48	0,389	1166
EHI_119470	1	876	4,804	1724
EHI_120570	1	8	0,071	1067
EHI_122410	1	19	0,094	1907
EHI_123820	1	8	0,05	1502
EHI_127100	1	178	1,162	1448
EHI_127710	1	18	0,115	1478
EHI_128460	1	2	0,027	713
EHI_129870	1	332	2,942	1067
EHI_131490	1	3051	24,116	1196
EHI_134140	1	4	0,029	1316
EHI_137910	1	42	0,124	3197
EHI_139390	1	223	1,519	1388
EHI_139430	1	205	0,96	2018

EHI_147680	1	139	1,133	1160
EHI_148460	1	604	5,475	1043
EHI_148530	1	150	0,954	1487
EHI_150150	1	30	0,731	388
EHI_151330	1	628	1,79	3317
EHI_152950	1	109	0,974	1058
EHI_153950	1	25	0,533	443
EHI_154170	1	12	0,149	759
EHI_158740	1	2	0,027	698
EHI_161300	1	13	0,233	527
EHI_163960	1	7	0,163	406
EHI_166160	1	1	0,02	467
EHI_173850	1	4	0,124	305
EHI_176480	1	43	0,561	725
EHI_184260	1	17	0,161	998
EHI_189090	1	1	0,009	1024
EHI_191510	1	27	0,189	1349
EHI_192250	1	364	3,427	1004
EHI_192600	1	61	0,705	818

EHI_194290	1	71	0,567	1184
EHI_198630	1	291	2,55	1079
EHI_199270	1	67	0,714	887
EHI_202250	1	1537	24,339	597

Figure S1: Three-dimensional prediction structure of EHI_016490 (CSP) from *Entamoeba histolytica* generated by I-TASSER.

Figure S2: Dendogram showing the relatedness between EHI_016490 and the BspA like proteins from *Entamoeba histolytica*. All 95 BspA like proteins expressed in trophozoites in *in vitro* culture were included in the phylogenetic tree, created by using ClustalX.

Figure S3: Three-dimensional prediction structure of the two most closely related BspA like proteins EHI_112030 (A) and EHI_151330 (B) from EHI_016490 (CSP) and the two most divergent BspA like proteins EHI_072070 (C) and EHI_152950 (D).

Figure S4: 10% polyacrylamide gel electrophoresis under reducing conditions, and a Western blot of *Entamoeba histolytica* crude lysates. Twenty µg of proteins were loaded. A polyclonal rabbit antiserum against two synthetic peptides from EHI_016490 detected bands at 45, 68 and 140 kDa. Pre-immune serum merely gave a low background signal.

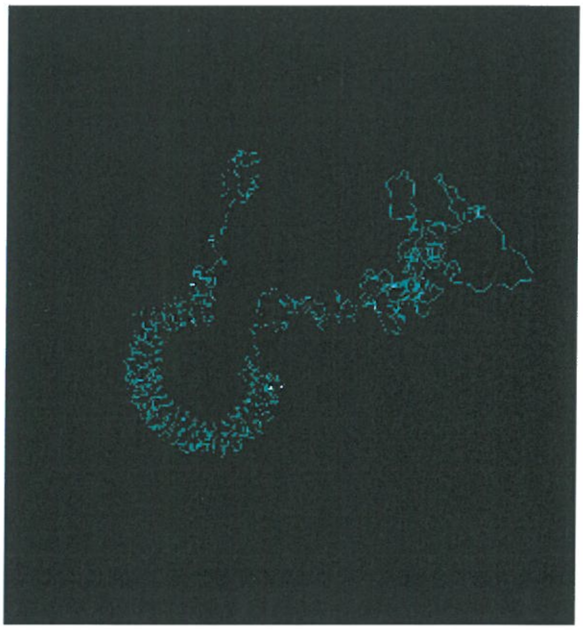

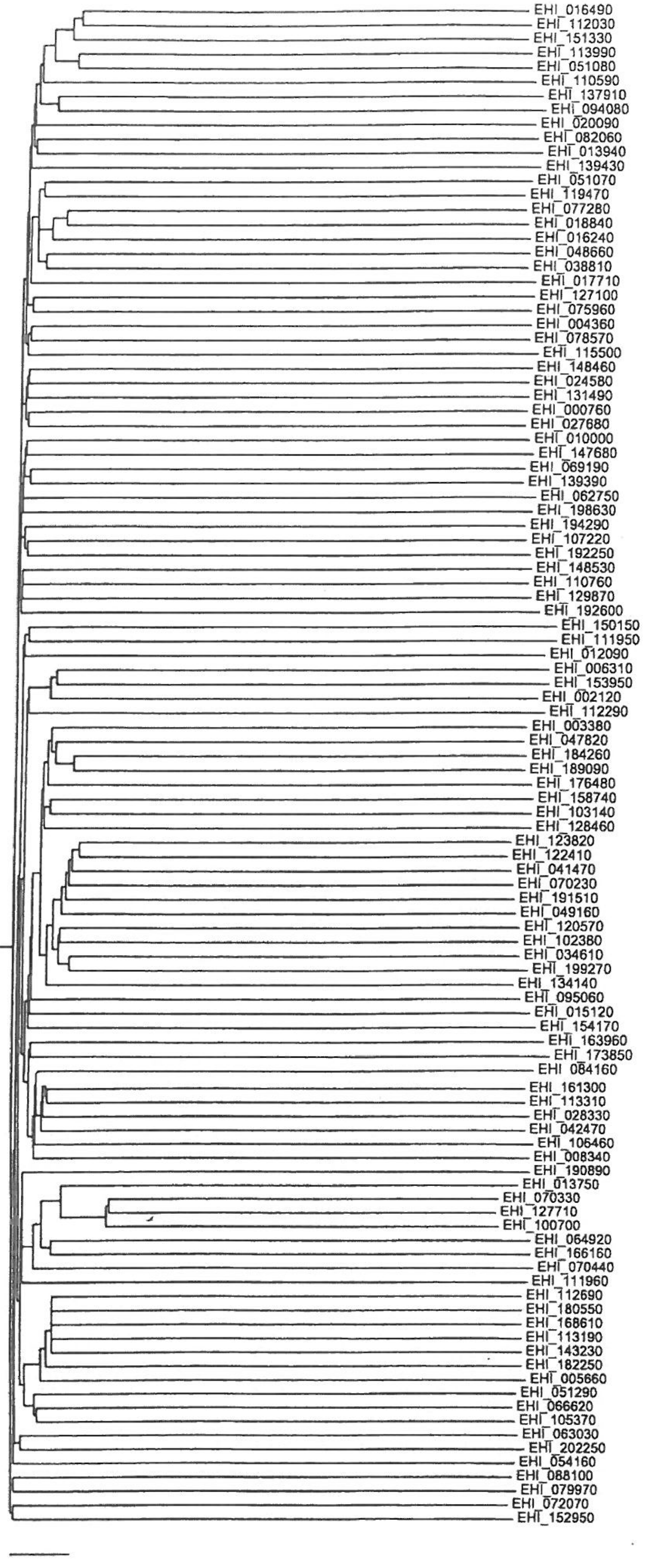
EHI_016490
EHI_112030
EHI_151330
EHI_113990
EHI_051080
EHI_110590
EHI_137910
EHI_094080
EHI_020090
EHI_082060
EHI_013940
EHI_139430
EHI_051070
EHI_119470
EHI_077280
EHI_018840
EHI_016240
EHI_048660
EHI_038810
EHI_017710
EHI_127100
EHI_075960
EHI_004360
EHI_078570
EHI_115500
EHI_148460
EHI_024580
EHI_131490
EHI_000760
EHI_027680
EHI_010000
EHI_147680
EHI_069190
EHI_139390
EHI_062750
EHI_198630
EHI_194290
EHI_107220
EHI_192250
EHI_148530
EHI_110760
EHI_129870
EHI_192600
EHI_150150
EHI_111950
EHI_012090
EHI_006310
EHI_153950
EHI_002120
EHI_112290
EHI_003380
EHI_047820
EHI_184260
EHI_189090
EHI_176480
EHI_158740
EHI_103140
EHI_128460
EHI_123820
EHI_122410
EHI_041470
EHI_070230
EHI_191510
EHI_049160
EHI_120570
EHI_102380
EHI_034610
EHI_199270
EHI_134140
EHI_095060
EHI_015120
EHI_154170
EHI_163960
EHI_173850
EHI_084160
EHI_161300
EHI_113310
EHI_028330
EHI_042470
EHI_106460
EHI_008340
EHI_190890
EHI_013750
EHI_070330
EHI_127710
EHI_100700
EHI_064920
EHI_166160
EHI_070440
EHI_111960
EHI_112690
EHI_180550
EHI_168610
EHI_113190
EHI_143230
EHI_182250
EHI_005660
EHI_051290
EHI_066620
EHI_105370
EHI_063030
EHI_202250
EHI_054160
EHI_088100
EHI_079970
EHI_072070
EHI_152950

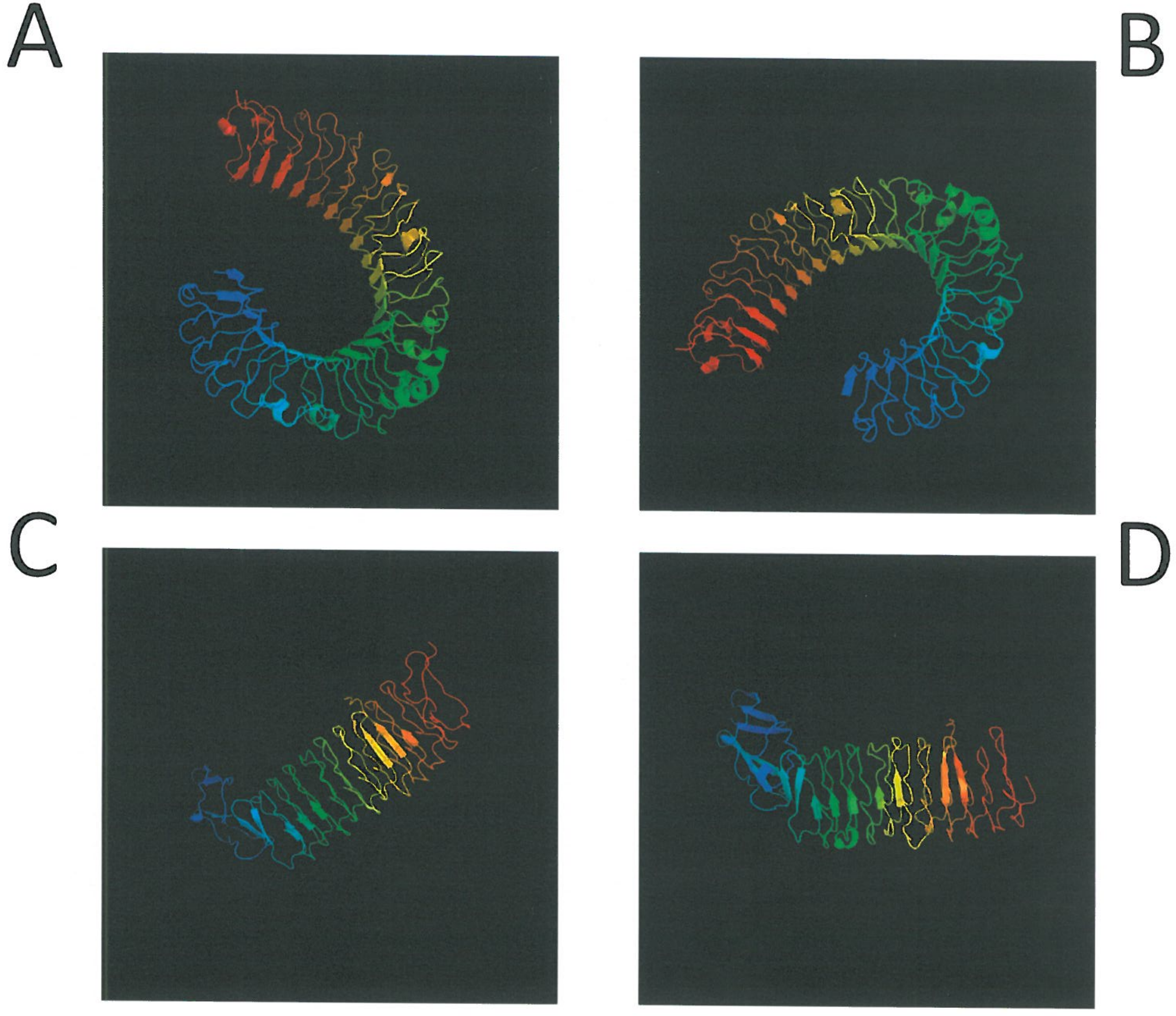
A
B
C
D

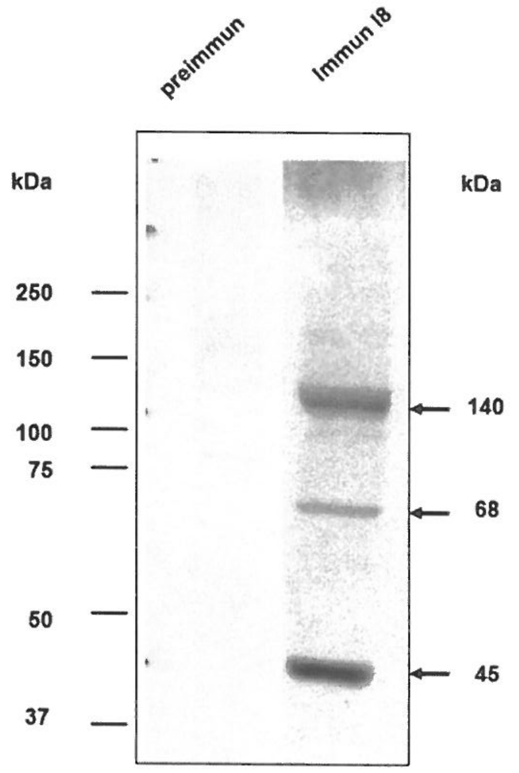
preimmun
Immun I8
kDa
250
150
100
75
50
37
kDa
140
68
45
